# Rocking during sleep reduces motor deficits and beta-amyloid levels in an Alzheimer’s mouse model

**DOI:** 10.1016/j.isci.2025.112036

**Published:** 2025-02-15

**Authors:** Luyan Zhang, Letizia Santoni, Nam Anh Ngo, Reyila Simayi, Eleonora Ficiará, Luisa de Vivo, Michele Bellesi

**Affiliations:** 1 School of Biosciences and Veterinary Medicine, University of Camerino, Camerino, Italy; 2 Center for Neuroscience, University of Camerino, Camerino, Italy; 3 Key Laboratory of Cold Chain Food Processing and Safety Control, Zhengzhou University of Light Industry, Zhengzhou, China; 4 School of Pharmacy, University of Camerino, Camerino, Italy

**Keywords:** Behavioral neuroscience, Biological sciences, Natural sciences, Neuroscience

## Abstract

Alzheimer’s disease (AD) is a neurodegenerative disorder characterized by cognitive decline, beta-amyloid plaques, and tau tangles. Growing evidence suggests a strong link between sleep disturbances and AD progression, with disrupted sleep exacerbating AD progression through increased beta-amyloid and tau accumulation. This relationship indicates that improving sleep quality could slow disease progression and mitigate its effects on the brain. We investigated whether vestibular stimulation (rocking) could mitigate AD pathology in 3xTg mice (*n* = 58, males). Starting in early adulthood (p60), mice underwent 12-h daily rocking during the light period for four months. Rocking increased non-rapid eye movement (NREM) sleep initially, although habituation reduced this effect over time. Despite habituation, rocking slowed motor decline and reduced beta-amyloid levels in the cerebral cortex and hippocampus. However, tau levels remained unaffected. In conclusion, our findings highlight the potential of non-pharmacological methods to enhance NREM sleep and modify disease trajectory in AD models.

## Highlights

• Rocking during sleep effectively increases the amount of NREM sleep in 3xTg mice
• Rocking during sleep mitigates motor deficits associated with disease progression
• Rocking during sleep reduces the brain levels of aggregating beta-amyloid

## Graphical abstract

**Figure:**
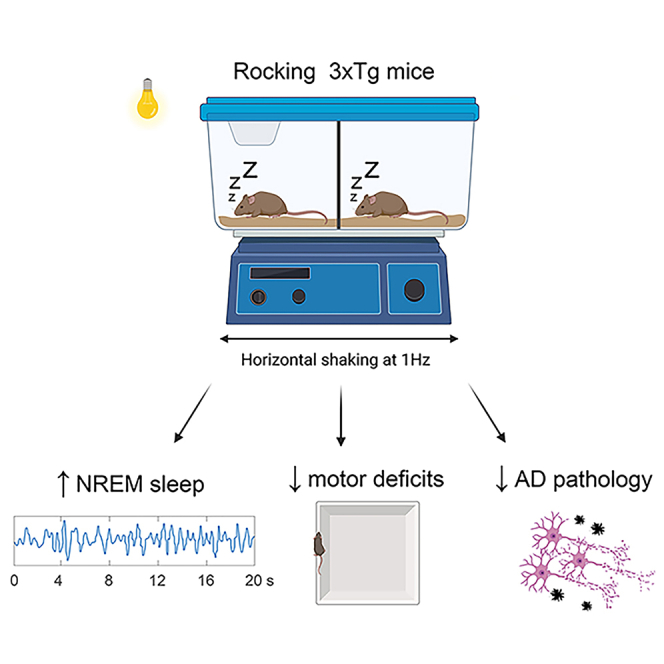

## Introduction

Alzheimer’s disease (AD) is a progressive neurological condition and the primary cause of dementia.^1^ Recent studies suggest a two-way relationship between AD pathology and sleep disturbances.^2^ Individuals with AD commonly experience disrupted sleep, marked by frequent nighttime awakenings and difficulty maintaining sleep. Conversely, chronically disturbed sleep raises the risk of AD and leads to significant increases in beta-amyloid and hyperphosphorylated tau levels, also driving tau pathology spreading in the brains of mice and humans.^3^^,^^4^^,^^5^^,^^6^ Moreover, the specific reduction in NREM slow wave activity (SWA), a marker of sleep homeostasis, has been associated with tau pathology in people with normal cognition or very mild cognitive impairment.^7^^,^^8^

While the exact mechanism linking sleep to the brain levels of beta-amyloid and hyperphosphorylated tau remains unclear, emerging research indicates that disrupted sleep compromises the glymphatic system’s function, a glial-dependent waste clearance pathway in the brain.^9^^,^^10^ This system clears soluble waste proteins and metabolic byproducts, including beta-amyloid and tau, preferentially during sleep. Consequently, insufficient NREM sleep may impair the glymphatic system function, thus limiting the removal of extracellular beta-amyloid and tau and favoring their accumulation.^9^ Another plausible mechanism involves the regulation of beta-amyloid and tau production by neuronal activity. Synaptic activity influences the extracellular release of these proteins, and since slow-wave sleep is characterized by reduced synaptic activity, it is possible that lower levels of beta-amyloid and tau result from decreased release rather than enhanced clearance.^11^

Regardless of the specific biological mechanism, these findings highlight the potential of improving NREM sleep as a therapeutic strategy to mitigate the accumulation and spread of pathological amyloid and tau, emphasizing its protective role in slowing the progression of AD.

Enhancing NREM sleep is commonly attempted through pharmaceutical interventions.^12^^,^^13^ However, drugs targeting GABAergic neurotransmission, such as gamma-hydroxybutyrate (GHB), gaboxadol, and tiagabine, though effective in increasing NREM sleep, pose risks of drug dependence, tolerance, and daytime side effects like somnolence. Moreover, they often fail to yield corresponding memory benefits in older adults and may even have amnestic effects.^14^^,^^15^^,^^16^^,^^17^ Alternatively, non-pharmacological methods have been explored, including magnetic or weak electric currents applied to the scalp during sleep, which can boost sleep slow waves.^18^^,^^19^ However, these methods, while effective, may be impractical or potentially hazardous with repeated application. Consequently, other studies have investigated the potential of enhancing sleep using more natural stimuli, such as sensory inputs, which may not carry the same practical limitations or potential risks associated with electrical/magnetic stimulation.^12^

In recent years, acoustic stimulation during sleep has been extensively studied as a method to enhance slow waves.^20^^,^^21^ This technique involves delivering acoustic stimuli either during the up-state of slow waves or continuously throughout slow-wave sleep. While effective, its limitations lie in the difficulty of fine-tuning sound intensity to align with sleep depth, minimizing unintended arousals and awakenings, and addressing individual variability in arousability and sensitivity to noise during sleep.^22^^,^^23^ Other sensory modalities, such as somatosensory and photic stimulation, have shown minimal efficacy in enhancing sleep.^24^^,^^25^

Vestibular stimulation, on the other hand, was one of the earliest techniques explored for its potential to promote sleep, likely due to the traditional belief that physical rocking aids in sleep induction, as seen in babies or hammock swinging. Research findings from studies in both humans and rodents have demonstrated that rocking not only facilitates sleep onset but also increases the duration of NREM sleep without impacting REM sleep.^26^^,^^27^^,^^28^

In this study, we investigated whether enhancing sleep through rocking could delay the progression of AD in a mouse model harboring familial AD mutations, characterized by both plaque and tangle pathology. We assessed the effects of rocking on sleep patterns, motor function, memory, and AD pathology over a four-month treatment period, starting in early adulthood.

## Results

### Rocking increases sleep duration, but its effect wanes over time

**Figure 1:**
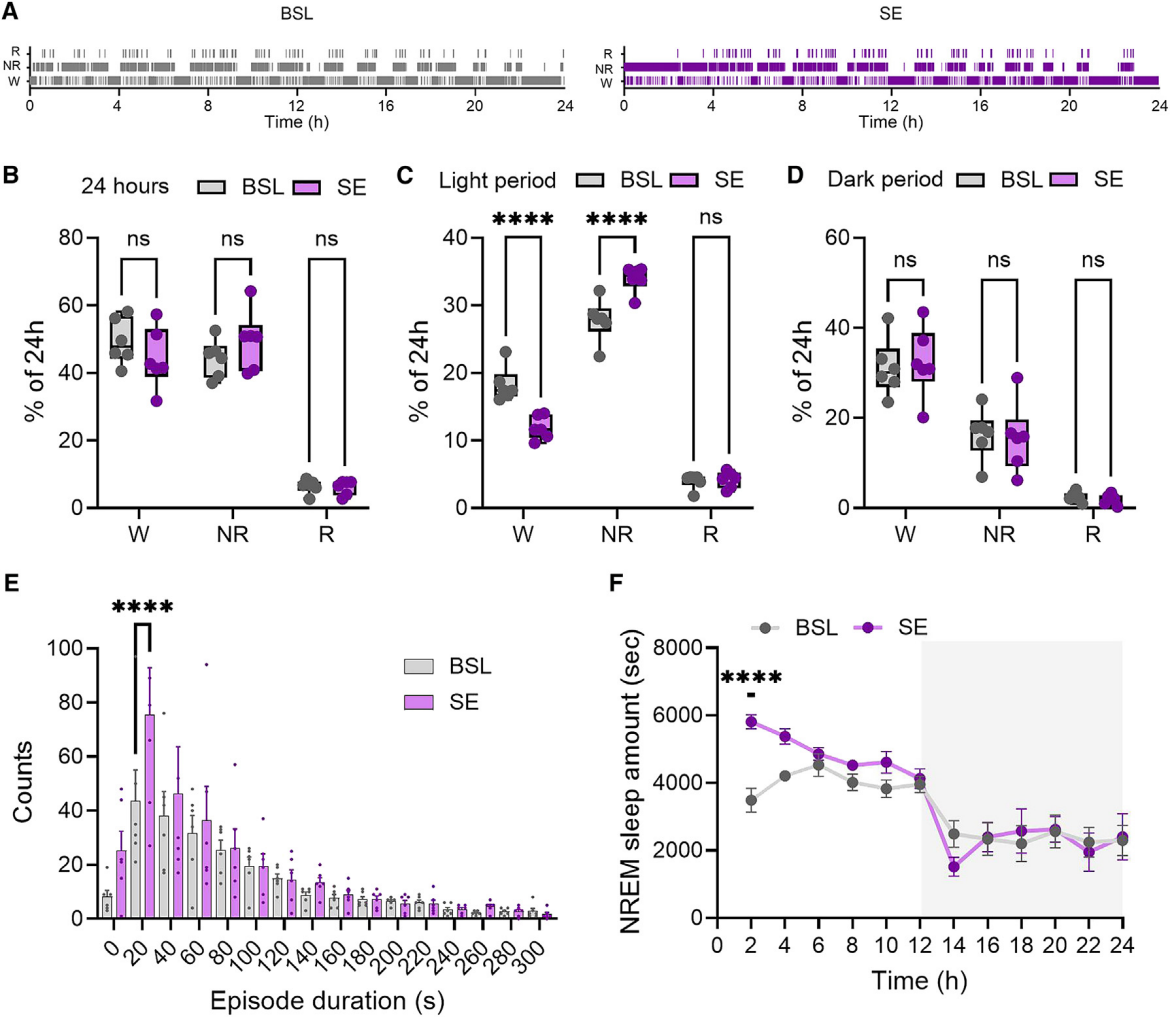
EEG-based analysis of rocking effects on sleep amount (A) Examples of baseline (BSL) and sleep enhancement (SE) hypnograms. (B) Distribution of wake (W), NREM sleep (NR), and REM sleep (R) amount in the 24h BSL (*n* = 6) and SE (*n* = 6). Dots represent individual mice; ns: not significant (hereafter). (C and D) Distribution of W, NR, and R amount in the light (C) and dark (D) period of BSL and SE. Dots represent individual mice. ∗∗∗∗*p* < 0.0001. (E) Frequency distribution of NREM sleep episodes for BSL and SE. Bars indicate mean ± sem and dots represent individual mice. ∗∗∗∗*p* < 0.0001. (F) Twenty-four–hour NREM time course for BSL and SE. Values are mean ± sem. Gray area indicates the dark period. ∗∗∗∗*p* < 0.0001.

Previous research on C57BL/6J wild type mice demonstrated that rocking at a 1Hz frequency during the light period extended NREM sleep duration at the expense of wakefulness.^27^ In an initial set of experiments, we aimed to verify the effectiveness of rocking in our 3xTg mice. To achieve this, we implanted EEG/EMG electrodes in six mice and recorded brain activity during a baseline period (no rocking) and an experimental day where they were rocked for 12 h during the light period. Analysis of the sleep architecture showed that rocking increased time spent in NREM sleep and reduced wake time, while having no effect on REM sleep. This effect was limited to the light period when rocking was active (NREM: *p* < 0.0001; REM: *p* = 0.99; Wake: *p* < 0.0001, Figures 1A–1C), as behavioral states during the dark period remained unaffected (NREM: *p* = 0.98; REM: *p* = 0.99; Wake: *p* = 0.92, Figure 1D). Further analysis of NREM episodes duration revealed that rocking predominantly increased the number of short to medium duration episodes (cluster 0–20 s: *p* = 0.0597; cluster 20–40 s: *p* < 0.0001, Figure 1E), indicating that rocking enhanced the propensity to fall asleep rather than extending the duration of long consolidate sleep episodes. Longer sleep periods were not affected. Analysis of NREM sleep time course demonstrated that the effect of rocking was more pronounced at the beginning of light period rather than at the end (bin 0-2 h: *p* < 0.0001; bin 2-4: *p* = 0.078, Figure 1F).

**Figure 2:**
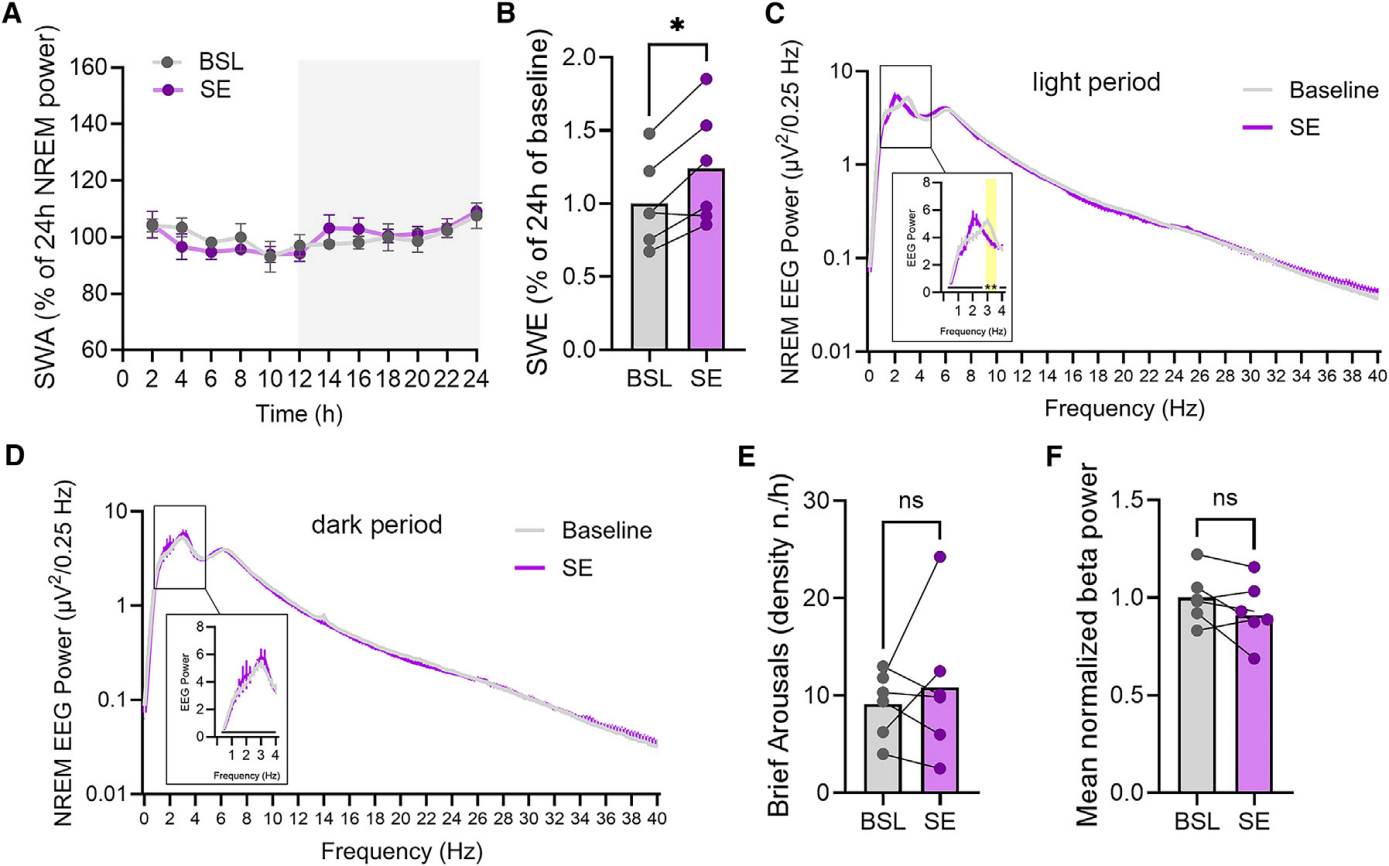
EEG-based analysis of rocking effects on EEG power and arousals (A) Twenty-four–hour time course of NREM SWA for BSL and SE. Values are mean ± sem. Gray area indicates the dark period. (B) Cumulative SWA (SWE) over the 12h light cycle in BSL and SE. Bars indicate mean, and dots represent individual mice. ∗*p* < 0.05. (C and D) Absolute NREM power spectrum of the light (C) and dark (D) period for BSL and SE. Insets highlight spectral differences between BSL and SE in the SWA frequency range. Yellow area in C indicates significant frequency bins (*p* < 0.05 corrected). Values are mean ± sem. (E) Density of brief arousals over the 12h light cycle for BSL and SE. Bars indicate mean, and dots represent individual mice. (F) Normalized beta (15-30Hz) power over the 12h light cycle. Bars indicate mean, and dots represent individual mice.

The increase in NREM sleep did not significantly influence averaged SWA (two-way ANOVA 24h: F (1, 60) = 1.474e-013 *p* > 0.99; two-way ANOVA only light period: F (1, 30) = 2.751 *p* = 0.11, Figure 2A), but it increased overall cumulative SWA over the 12h of rocking (Slow wave energy [SWE], *p* = 0.011, Figure 2B). Comparing the NREM sleep power spectrum between baseline and rocking condition, we found that rocking induced a shift in the SWA peak toward a frequency of 2 Hz (two-way ANOVA NREM: F (160, 1600) = 2.16 *p* < 0.0001; Sidak’s multiple comparisons test: *p* = 0.26 [bin 3–3.25 Hz]; *p* = 0.24 [bin 3.25–3.5 Hz], Figure 2C). This shifting effect was more obvious when the analysis was restricted to the SWA frequency range (0.5–4 Hz) (two-way ANOVA: F (14, 140) = 4.691, *p* < 0.0001; Sidak’s multiple comparisons test: *p* = 0.0285 [bin 3–3.25 Hz]; *p* = 0.026 [bin 3.25–3.5 Hz], Figure 2C inset). Notably, this effect was absent during NREM epochs from the dark phase, when rocking was inactive (two-way ANOVA 0.5–4 Hz: F (4, 140) = 0.34, *p* = 0.99; no significant bins, Figure 2D). A similar but less pronounced trend was observed in REM sleep and wake states, but the shifting effect did not reach statistical significance in either vigilance state (two-way ANOVA REM: F (160, 1600) = 0.74, *p* = 0.99; no significant bins; two-way ANOVA Wake: F (160, 1600) = 0.82, *p* = 0.94; no significant bins, Figure S1). Finally, to evaluate whether vestibular stimulation induced arousals or high-frequency activity during sleep, we assessed the density of brief arousals and analyzed beta power (15–30 Hz) during NREM sleep. Neither the density of brief arousals (*p* = 0.53; Figure 2E) nor the NREM beta activity (*p* = 0.19; Figure 2F) were significantly affected by rocking.

**Figure 3:**
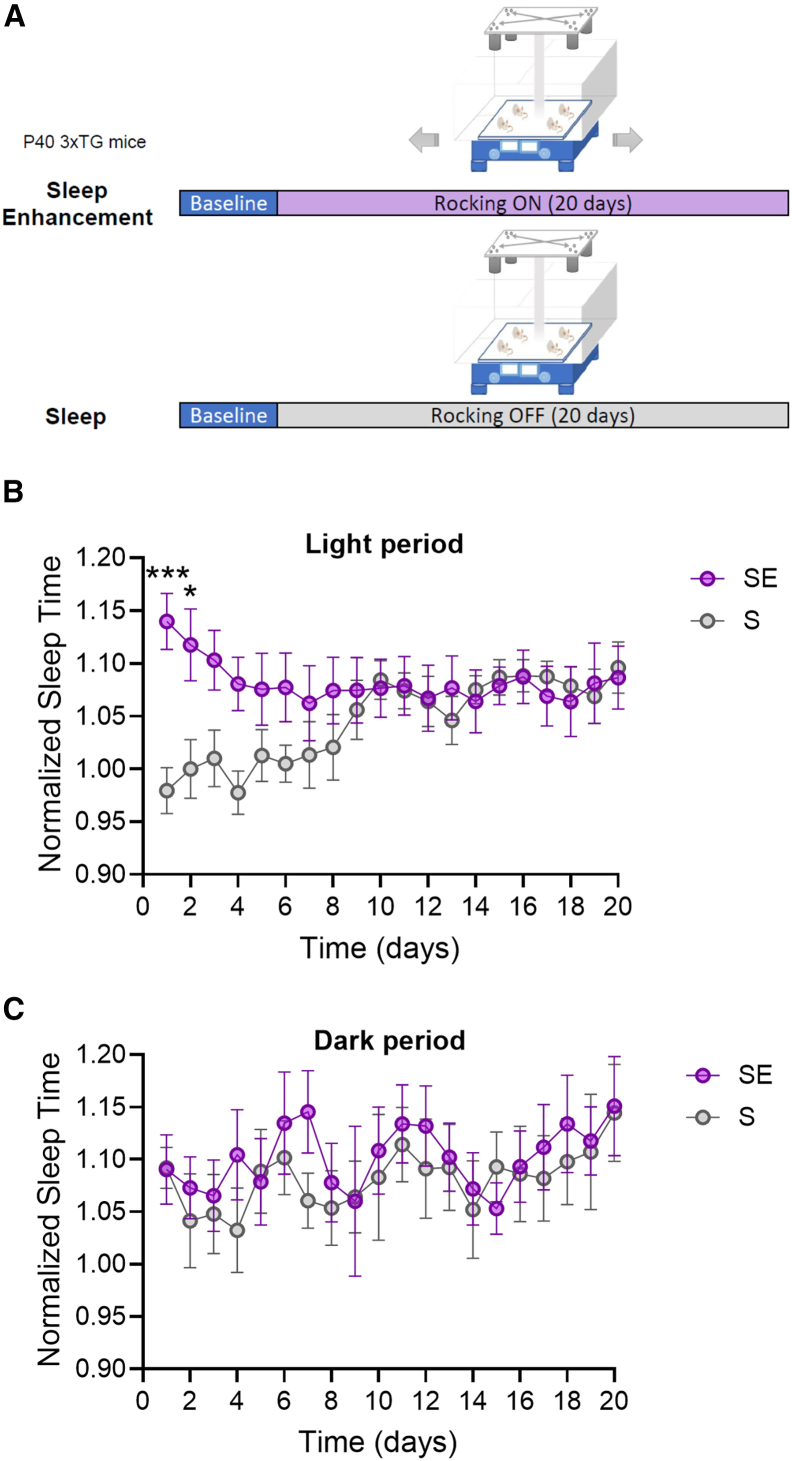
Motion-based analysis of rocking effects on sleep amount in the first experiment (A) Experimental design of Exp 1. (B and C) Sleep amount over the 20 days of observation period for light (B) and dark (C) period. Sleep amount is normalized to baseline mean sleep duration (Sleep [S], *n* = 8; SE, *n* = 8). Values are mean ± std. ∗*p* < 0.05; ∗∗∗*p* < 0.001.

In chronic experiments, we utilized sleep and wake quantification based on motion detection analysis to avoid keeping the mice implanted and tethered for extended periods, thereby reducing animal stress and the risk of inflammation and tissue reactions associated with electrode implants. We initially carried out a first experiment (Exp 1) to establish the optimal duration of the stimulation. In this experiment mice were rocked for 12 h during the light period for 20 consecutive days starting at postnatal day (p)40 (Figure 3A). Exposure to rocking increased the amount of sleep time in the first days, but this effect progressively faded off over the course of the stimulation, likely due to habituation (two-way ANOVA: F (1, 280) = 17.66 *p* < 0.0001; Figure 3B). Sleep amount during the dark period did not differ between the two groups (two-way ANOVA: F (1, 280) = 2.547 *p* = 0.1116; Figure 3C).

**Figure 4:**
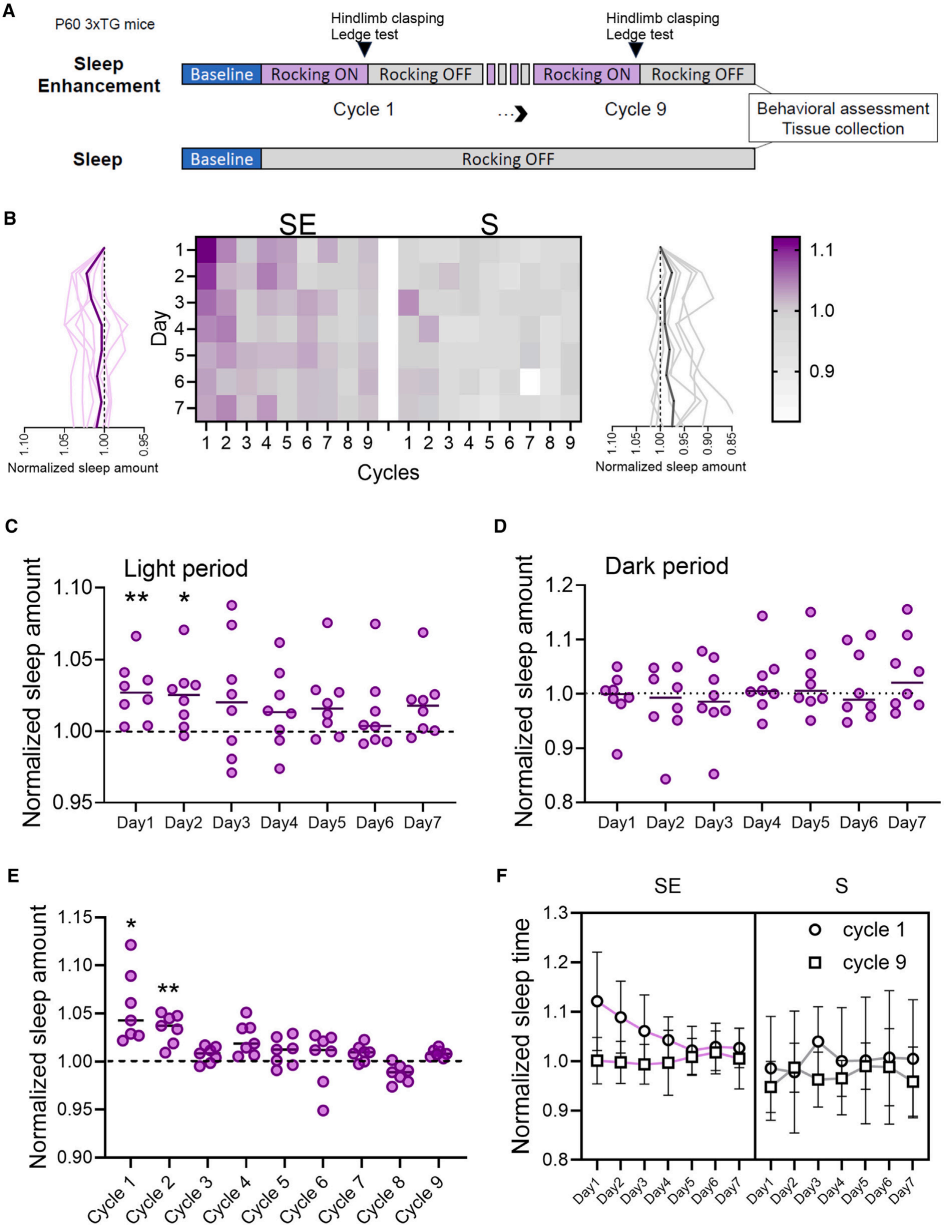
Motion-based analysis of rocking effects on sleep amount (A) Experimental design of Exp 2 (SE, *n* = 8, S = 8). (B) Heat diagram showing normalized sleep amount for SE (left) and S (right) mice across days and cycles during light period. Graphs on side represent averaged (thick line) and single mouse trends (thin lines) across days. Scale bar shows normalized sleep amount. (C) Averaged quantification of sleep amount over the 7 days of rocking period (light period only) for SE mice. Values are relative to mean baseline values (dashed line). Each dot represents an individual mouse. ∗*p* < 0.05; ∗∗*p* < 0.01. (D) Averaged sleep amount over 7 days of rocking period (dark period only) for SE mice. (E) Averaged quantification of sleep amount over the 7 days of rocking period (light period only) for SE mice. Values are relative to mean baseline values (dashed line). Each dot represents an individual mouse. ∗*p* < 0.05; ∗∗*p* < 0.01. (F) Sleep amount for cycle 1 and 9 over 7 days of rocking period (light period only) averaged across SE and S mice. Values are mean ± std.

In following experiments (Exp 2), we modified the stimulation paradigm by implementing a block design, alternating between a week of stimulation and a week of no stimulation to contrast habituation. This pattern was repeated for 9 cycles over a total of 18 weeks, during which we continuously monitored sleep and wake via motion detection (Figure 4A). Analysis of sleep duration during the stimulation weeks for the SE group revealed an increase compared to baseline, while sleep duration remained stable in the control S group (Figure 4B). The effect of rocking was more pronounced initially and diminished over the course of the stimulation week (Day1 vs. BSL: *p* = 0.0069; Day2 vs. BSL: *p* = 0.019; Figure 4C). Of note, rocking did not affect sleep duration during the dark phase (RM-1way ANOVA: F (1.989, 13.92) = 0.95 *p* = 0.4096; Figure 4D).

Moreover, we examined the longitudinal impact of rocking on sleep duration across the nine cycles. Our analysis revealed that the influence of rocking was most pronounced initially and gradually diminished in parallel with aging and disease advancement (RM-1wayANOVA: F (1.732, 10.39) = 7.995 *p* = 0.0094 Figures 4E and 4F).

**Figure 5:**
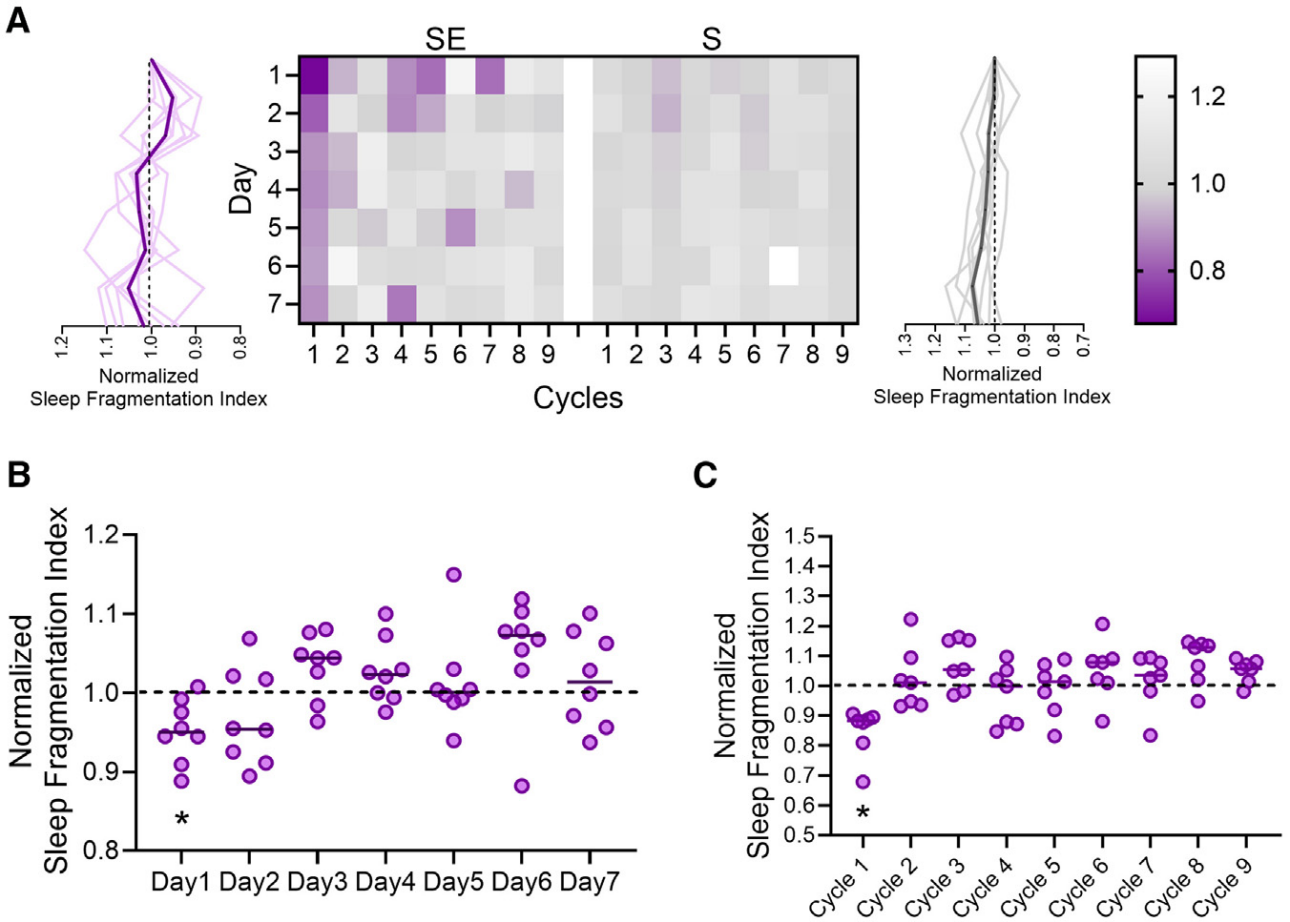
Motion-based analysis of rocking effects on sleep fragmentation (A) Heat diagram showing normalized sleep fragmentation index for SE (*n* = 8, left) and S (*n* = 8, right) mice across days and cycles during light period. Graphs on side represent averaged (thick line) and single mouse trends (thin lines) across days. Scale bar shows normalized sleep fragmentation index. (B) Averaged quantification of sleep fragmentation index over the 7 days of rocking period (light period only) for SE mice. Values are relative to mean baseline values (dashed line). Each dot represents an individual mouse. ∗*p* < 0.05. (C) Averaged quantification of sleep fragmentation index over the 9 cycle of rocking period (light period only) for SE mice. Values are relative to mean baseline values (dashed line). Each dot represents an individual mouse. ∗*p* < 0.05.

In addition to sleep amount, we quantified sleep fragmentation by counting sleep-to-wake transitions from the motion activity patterns. This analysis showed that rocking not only prolonged sleep duration but also reduced sleep fragmentation in SE mice (Figure 5A). The pattern of this effect mirrored the trend in sleep duration, being most noticeable at the start of the stimulation week (RM-1wayANOVA: F (2.873, 20.11) = 3.282 *p* = 0.0436) and in the initial cycles (RM-1wayANOVA: F (3.024, 18.15) = 5.155 *p* = 0.0093; Figures 5B and 5C).

Thus, rocking enhanced both sleep duration and stability. However, this effect was more pronounced initially and diminished gradually over time, both within individual cycles and across successive cycles of stimulation.

### Rocking delays motor behavior impairment

**Figure 6:**
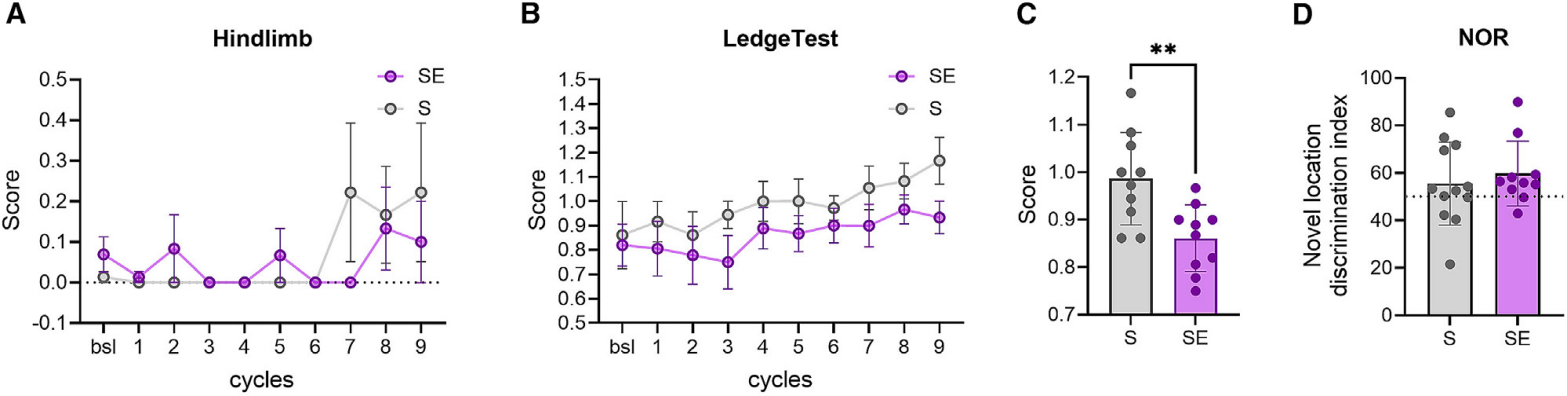
Behavioral effects of rocking (A) Averaged scores at the hindlimb test carried out at each cycle (1 → 9). (SE, *n* = 12, S, *n* = 12). Values are mean ± sem. (B) Averaged score at the ledge walking test carried out at each cycle (1 → 9). (SE, *n* = 12, S, *n* = 12). Values are mean ± sem. (C) Averaged scores at the ledge walking test across cycles (*n* = 9). (D) Individual scores of the novel object discrimination index at the novel object recognition test carried out after cycle 9. Dots represent individual mice (SE, *n* = 10, S, *n* = 12), while the dashed line shows when mice spent equal time investigating the old and new objects. In all bar graphs, lines represent mean ± std. ∗∗*p* < 0.01.

Throughout the sleep manipulation experiments, mice underwent biweekly behavioral evaluations using hindlimb clasping and ledge tests. These assessments evaluate motor function and coordination and are recognized as indicators of neurodegenerative diseases including AD. Analysis of the scores from these tests over time revealed a decline attributed to disease progression. Notably, scores at the hindlimb clasping test showed no significant effect between S and SE (two-way ANOVA: F (1, 210) = 0.2307 *p* = 0.63, Figure 6A), while SE mice outperformed S mice at the ledge walking, a test which requires significant motor coordination (two-way ANOVA: F (1, 210) = 9.53 *p* = 0.0023, Figure 6B; unpaired t-test: *p* = 0.004, Figure 6C). The novel object recognition test at the final assessment did not show any significant difference between the groups (unpaired t-test: *p* = 0.5375, Figure 6D).

Thus, rocking delayed the progression of motor impairment as evaluated at the ledge walking test but had no effect on learning and memory.

### Rocking reduces beta-amyloid levels

**Figure 7:**
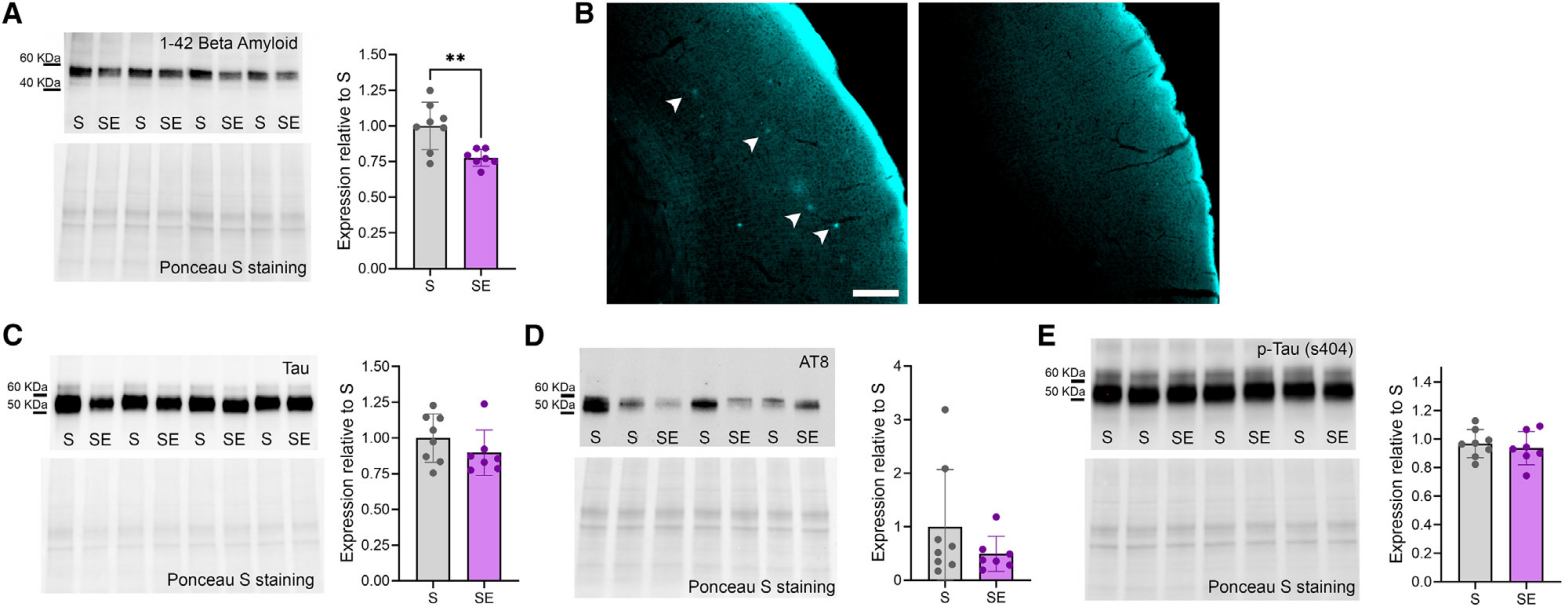
Quantification of beta-amyloid and tau levels in the cerebral cortex (A) Representative immunoblot bands stained against beta-amyloid 1–42 and its expression levels in the cerebral cortex of S (*n* = 8) and SE (*n* = 7) mice. ∗∗*p* < 0.01. (B) Representative microscopy fields of cerebral cortex stained with Methoxy-X04 and acquired from a 12-month-old (left) and an S (right) 3xTg mouse. Note that, unlike the aged mouse (left) used as a positive control, the S mouse (right) shows no beta-amyloid plaques (arrow heads). Scale bar: 200 μm. (C) Representative immunoblot bands stained against tau and its expression levels in the cerebral cortex of S (*n* = 8) and SE (*n* = 7) mice. (D) Representative immunoblot bands stained against AT8 and its expression levels in the cerebral cortex of S (*n* = 8) and SE (*n* = 7) mice. (E) Representative immunoblot bands stained against p-tau (ser 404) and its expression levels in the cerebral cortex of S (*n* = 8) and SE (*n* = 7) mice. In all graphs, each dot represents an individual mouse, bars represent mean ± std.

**Figure 8:**
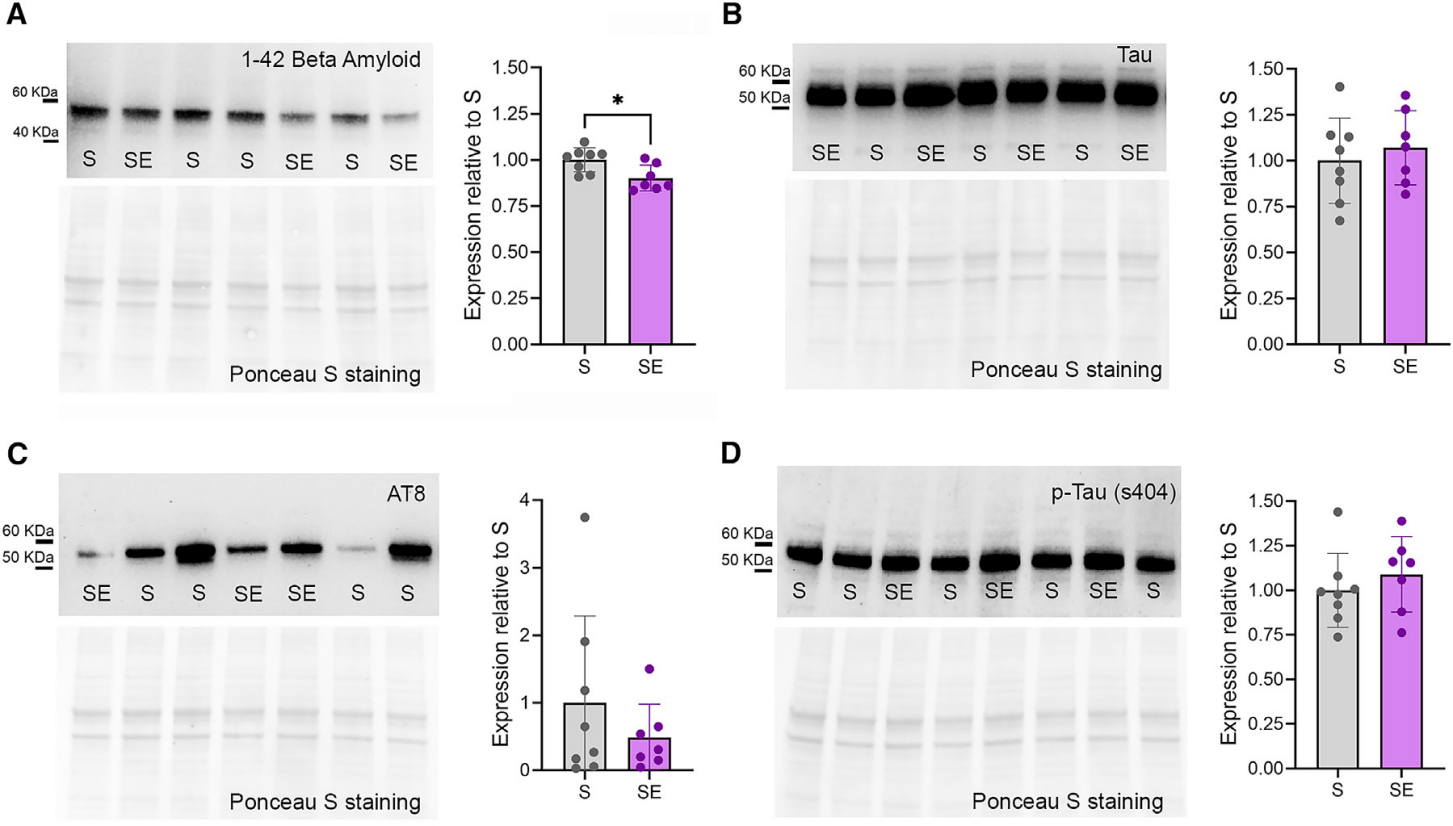
Quantification of beta-amyloid and tau levels in the hippocampus (A) Representative immunoblot bands stained against beta-amyloid 1–42 and its expression levels in the hippocampus of S (*n* = 8) and SE (*n* = 7) mice. ∗*p* < 0.05. (B) Representative immunoblot bands stained against tau and its expression levels in the hippocampus of S (*n* = 8) and SE (*n* = 7) mice. (C) Representative immunoblot bands stained against AT8 and its expression levels in the hippocampus of S (*n* = 8) and SE (*n* = 7) mice. (D) Representative immunoblot bands stained against p-tau (ser 404) and its expression levels in the hippocampus of S (*n* = 8) and SE (*n* = 7) mice. In all graphs, each dot represents an individual mouse, bars represent mean ± std.

To assess AD pathology, we conducted immunoblot analyses to measure beta-amyloid and tau levels in cerebral cortex and hippocampus samples from SE and S mice. This analysis revealed a significant decrease in beta-amyloid levels in SE compared to S mice (unpaired t-test, cortex: *p* = 0.005; hippocampus: *p* = 0.0142 Figures 7A and 8A). However, examination of beta-amyloid plaques showed no noticeable difference between the two groups, as formations suggestive of plaques were very rare or absent in most of the SE and S mice (Figure 7B). Total tau levels remained largely unchanged between the groups (unpaired t-test, cortex: *p* = 0.2474; hippocampus: *p* = 0.5457, Figure 7C; 8B). In addition, the levels of AT8 and phosphorylated tau at serine 404 showed no significant differences between S and SE (unpaired t-test, cortex: AT8, *p* = 0.26; hippocampus: *p* = 0.337, Figures 7D and 8C; cortex: p-Tau s404, *p* = 0.58; hippocampus: *p* = 0.423 Figures 7E and 8D).

**Figure 9:**
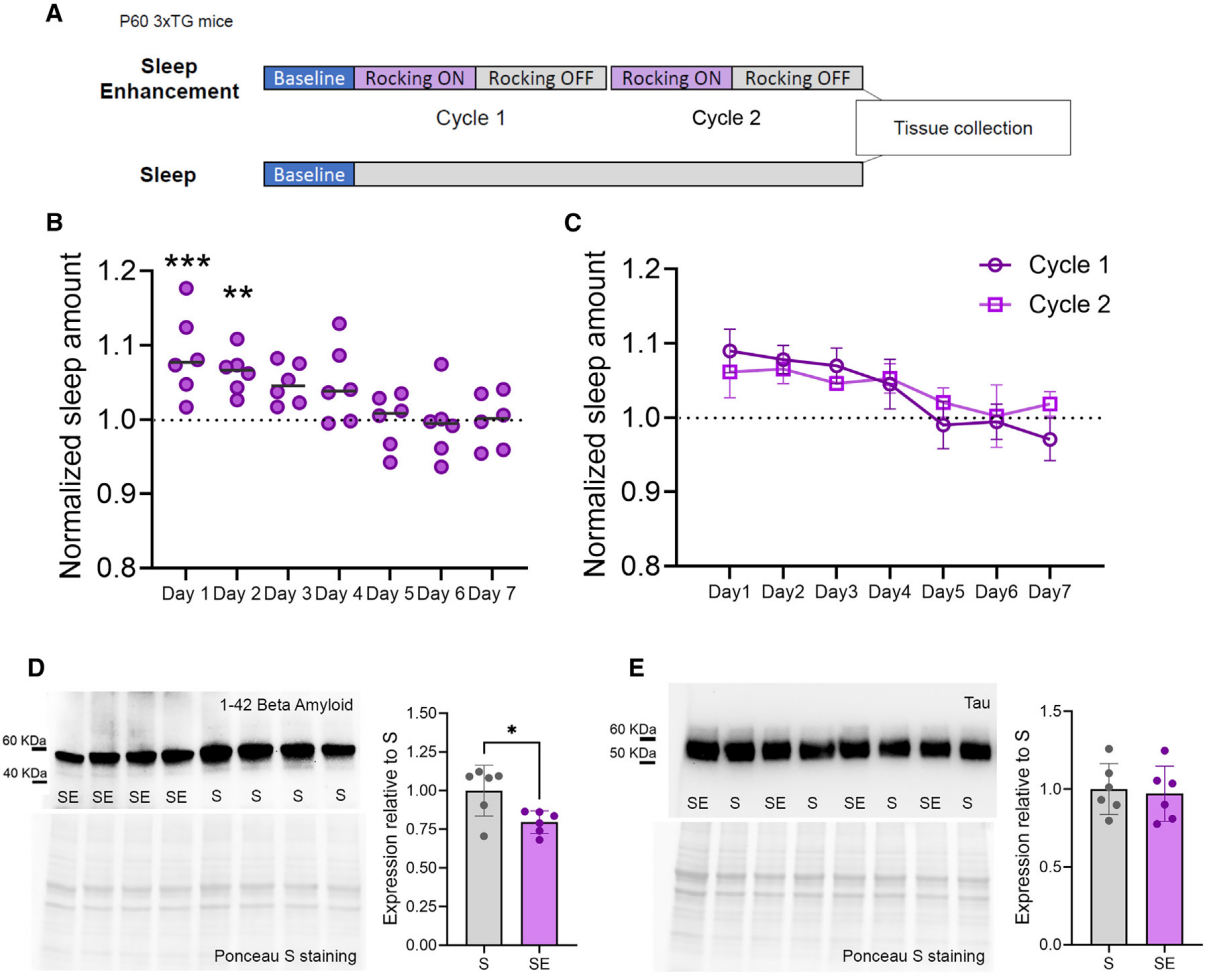
Behavioral and molecular effects after two cycles of rocking (A) Experimental design of Exp 3 (S, *n* = 6, SE = 6). (B) Averaged quantification of sleep amount over the 7 days of rocking period (light period only) for SE mice. Values are relative to mean baseline values (dashed line). Each dot represents an individual mouse. ∗∗*p* < 0.01; ∗∗∗*p* < 0.001. (C) Sleep amount for cycle 1 and 2 over 7 days of rocking period (light period only) averaged across SE and S mice. Values are mean ± std. (D) Representative immunoblot bands stained against beta-amyloid 1–42 and its expression levels in the cerebral cortex of S (*n* = 6) and SE (*n* = 6) mice. ∗*p* < 0.05. (E) Representative immunoblot bands stained against tau and its expression levels in the cerebral cortex of S (*n* = 6) and SE (*n* = 6) mice. In D-E, each dot represents an individual mouse, bars represent mean ± std.

In a separate group of mice, we repeated the chronic experiment (Exp 3), assessing beta-amyloid and tau levels at the end of the second cycle (Figure 9A), when the effects of rocking on promoting sleep were more evident and less influenced by habituation. As in the longer experimental design, rocking primarily improved sleep during the first part of the week, with no change in effectiveness during the second cycle compared to the first (Figures 9B and 9C). Cortical beta-amyloid levels at the end of the second cycle confirmed that rocking reduced beta-amyloid accumulation (unpaired t-test, beta-amyloid, *p* = 0.0192, Figure 9D), while analysis of total tau showed no effect at this stage (unpaired t-test, total tau, *p* = 0.77, Figure 9E).

## Discussion

In this study, we demonstrated that rocking 3xTg mice during their sleep period increases NREM sleep duration. However, chronic sleep monitoring revealed that this effect diminishes over time as the mice become habituated to the rocking and as the disease progresses. Despite this, rocking reduced the deterioration of motor behavior over time, which typically occurs in this mouse model. Additionally, rocking was associated with reduced cerebral cortex and hippocampus levels of beta-amyloid, although it did not significantly affect the expression of total tau and phosphorylated tau.

We found that rocking at 1 Hz during the light period, when the mice normally sleep, led to an increase in time spent in NREM sleep. As observed previously in wild-type animals, this effect occurred at the expense of waking time.^27^ Further EEG analysis showed that rocking promoted transitions to short-to medium-duration sleep episodes rather than extending long consolidated sleep, suggesting that, as in humans, rocking facilitates the falling asleep process. The overall increased NREM duration did not change the average amount of SWA, indicating that rocking, unlike other sensory stimulations during sleep (e.g., acoustic stimulation),^29^ did not significantly influence sleep intensity. However, when SWA was computed over time, a cumulative effect of rocking was evident due to the greater overall sleep duration. The lack of a measurable effect on average SWA levels is consistent with findings reported by Kompotis et al. (2019)^27^ in mice and Perrault et al. (2019)^28^ in humans, which suggest that while vestibular stimulation at specific frequencies facilitates sleep initiation, its capacity to enhance SWA is limited. The underlying mechanisms remain unclear but may be related to the ability of the vestibular stimulation to entrain slow oscillations rather than increasing the recruitment of neurons into synchronous oscillatory activity, as observed, for instance, with acoustic stimulation.^12^ Acoustic stimuli during NREM sleep appear to robustly engage arousal systems, promoting widespread synchronization of bistable neuronal populations, thereby increasing slow-wave amplitude and overall SWA.^12^^,^^30^^,^^31^ In contrast, vestibular stimulation may exert its effects on sleep through distinct neuronal pathways and a minor involvement of the wake promoting systems.^26^

Consistent with the hypothesis that vestibular stimulation promotes entrainment of neuronal oscillatory activity, we observed that rocking induced a general slowing of brain rhythms. This effect was most pronounced during NREM sleep, where the power spectrum peak shifted to lower frequencies, centering around 2 Hz. Importantly, this effect was absent when analyzing NREM epochs recorded during the dark period, when rocking was inactive, indicating that the observed changes were specifically driven by the rocking stimulus. A similar but less pronounced slowing effect was observed in other vigilance states. This suggests that rocking entrains brain rhythms, with the impact being most evident during NREM sleep, when slow oscillations dominate. Interestingly, the NREM power spectrum peak shifted to approximately 2 Hz rather than 1 Hz, that is the rocking frequency. This may be related to the fact that the vestibular stimulus is encoded by the brain at approximately twice that frequency of rocking.^27^

In this study, we did not utilize a closed-loop procedure to deliver sensory stimulation exclusively during deep NREM sleep, as is commonly done with acoustic stimulation in human studies.^21^ Consequently, we do not know whether applying rocking in such a manner would have produced a stronger effect. However, based on our current data and findings from other studies, it appears that rocking primarily facilitates sleep onset rather than affecting the intensity of preexisting sleep episodes.^27^^,^^28^

Also, we did not explore other sensory modalities to enhance sleep, leaving open the possibility that they might yield similar results. For instance, in rodents, olfactory stimulation has been shown to induce slow waves.^32^ However, this method proved ineffective in humans, likely due to the limited impact of olfactory stimuli on human thalamocortical networks.^25^^,^^33^ Similarly, studies investigating somatosensory and visual stimulation have found that somatosensory inputs during NREM sleep induces only minor changes, while visual stimulation appears largely ineffective in enhancing sleep.^24^^,^^25^ Interestingly, exposing mice to a warm environment has shown promise in facilitating sleep.^34^ A recent study revealed that this approach not only increased the proportion of diurnal slow-wave sleep but also reduced hippocampal beta-amyloid levels in a mouse AD model.^35^ These findings are consistent with the evidence linking optogenetically induced slow oscillations to reduced amyloid plaque formation,^36^ further highlighting the potential neuroprotective benefits of slow oscillatory activity typically observed in NREM sleep.

We found that the effect of rocking on sleep is not constant over time. Even within a single 12-h period, the sleep-promoting effect of rocking decreases over time, demonstrating a clear habituation phenomenon. This habituation was further observed over multiple days of stimulation. Habituation to sensory stimulation has a long history in research.^37^ Early studies by Sokolov demonstrated that repetitive patterns of stimulation can induce rapid habituation of the response.^38^ This mechanism has been attributed to the reticular formation and is due to a progressive reduction of synaptic efficacy within its circuits.^39^ In the case of rocking, habituation could also result from neural adaptation of the vestibular pathways.^40^ In our study, we attempted to overcome habituation by implementing a block design, with weeks of stimulation alternated with weeks without stimulation. This approach reduced habituation, as the first days of a new cycle consistently showed better sleep promoting effects than the remaining days. However, the overall sleep promoting effect of rocking declined irreparably over several weeks. It is possible that this decline in effectiveness is not solely due to habituation but also to the progression of the disease and a decrease in overall sleep quality in this mouse model.

Despite the inconsistent effect of rocking on sleep throughout the experiment, we observed some beneficial effects of rocking on motor performance. Motor coordination is one of the early deficits in 3xTg mice and is commonly assessed to evaluate disease progression over time.^41^ In male mice motor deficit is also more pronounced than in female mice.^42^^,^^43^ We used the ledge test, which is very sensitive, easy to implement, and can be administered multiple times, to chronically monitor motor performance.^44^ Previous studies have shown that chronic sleep disruption can impair motor coordination, as assessed by the ledge test in a mouse model of AD.^5^ In our study, rocking was associated with reduced motor performance deterioration, suggesting a potential link between this intervention and better preservation of motor function. While improved sleep may contribute to better motor performance, it is also possible that the rocking stimulus directly influenced motor function independently. We did not find any effect on the novel object recognition task. This lack of effect may be due to the relatively young age of the animals at testing (P130-140). Cognitive deficits in these mice typically become more apparent around six-eight months of age,^45^ which could explain the absence of noticeable learning and memory impairment at the time of testing.

In association with improved motor function, we found that rocking decreased levels of beta-amyloid in the cerebral cortex and hippocampus. This reduction was notable even in the absence of detectable plaques, likely due to the young age of the mice at the time of sacrifice.

It is important to mention that the habituation observed in our experiments raises the possibility that the molecular effects we identified on beta-amyloid at the end of the study were not solely dependent on the sleep-enhancing properties of rocking but could also be directly influenced by the vestibular stimulation itself. To explore this, we conducted an additional experiment with a design limited to the first two cycles, a period when the sleep-enhancing effects of rocking were still evident. Our results confirmed that rocking reduced beta-amyloid levels at this earlier stage. While these findings do not entirely rule out a direct contribution of rocking to the observed effects, they support a link between enhanced sleep and beta-amyloid reduction. In addition, these results align with recent evidence demonstrating that sleep induced by mechanosensory stimulation can mitigate AD pathology in drosophila.^46^

Numerous studies have linked sleep, or the lack thereof, to beta-amyloid level dynamics and accumulation in both mouse models and humans.^8^^,^^47^^,^^48^^,^^49^ Research has shown that sleep, particularly NREM sleep, facilitates the clearance of beta-amyloid from the brain.^9^ During sleep, the glymphatic system is more active, enhancing the removal of beta-amyloid. Conversely, sleep deprivation or disruption has been associated with increased beta-amyloid accumulation.^10^ Chronic sleep disturbances can impair the efficiency of the glymphatic system, leading to a buildup of beta-amyloid, which can further disrupt sleep creating a vicious cycle.^10^ Although recent evidence has challenged the role of sleep in clearing the extracellular space from potentially accumulating toxins,^50^ the evidence supporting sleep in preventing beta-amyloid accumulation remains robust.^2^ Consistently with these findings, it is possible to hypothesize that enhancing sleep via rocking may help reduce beta-amyloid levels in the brain, suggesting a potential link between improved sleep and the regulation of beta-amyloid. However, we cannot ascertain whether this effect is mediated by improved clearance through a more efficient glymphatic system or by reduced production of beta-amyloid.

Recent research indicates that chronic sleep restriction significantly increases levels of hyperphosphorylated tau and exacerbates tau pathology progression in both mice and humans.^5^^,^^6^ Specifically, reductions in slow wave sleep have been associated with tau pathology in individuals with normal cognitive function or mild cognitive impairment.^7^^,^^8^ However, our findings revealed that rocking, whether in the short (Exp 3) or long (Exp 2) experimental design, did not affect levels of total tau or phosphorylated tau proteins. One possible explanation for this lack of effect on tau pathology may reside in the animal model used in our study. 3xTg mice typically develop both plaque and tangle pathology, with detectable accumulations of conformationally altered and hyperphosphorylated tau in the hippocampus occurring between 12 and 15 months of age.^51^ In contrast, our mice were assessed for tau pathology at a much younger age. It is plausible that the absence of an observable effect of rocking on tau pathology in our study could be due to the fact that tau pathology had not yet developed at the age when the mice were tested. Another possibility is that modifications in tau protein levels, rather than beta-amyloid deposition, may be more sensitive to changes in sleep intensity. Although direct experimental evidence for this hypothesis is lacking, data from a cohort of human subjects who underwent PET imaging and EEG sleep recordings revealed an inverse relationship between NREM SWA and AD pathology.^7^ NREM SWA decreased as evidence of beta-amyloid deposition and tau accumulation increased. Notably, this relationship was stronger with tau pathology than with beta-amyloid pathology,^7^ suggesting that enhancing SWA, rather than extending NREM sleep duration, may be a more effective approach to positively affect tau pathology.

In conclusion, this study showed that vestibular stimulation via rocking can enhance sleep and modify the trajectory of the disease in a mouse model of AD. These findings also confirm that vestibular stimulation during sleep may represent a viable way to offer neuroprotection in AD.

### Limitations of the study

In this work, we focused on enhancing sleep during young adulthood, a period when mice still exhibit relatively good sleep quality. It remains unclear whether this method of sleep enhancement would be equally effective in older mice or lead to similar outcomes. Moreover, we studied only male mice to reduce variability that could arise from the potential effects of hormonal fluctuations on sleep. As a result, it is unclear whether the observed effects are also applicable to female mice.

Also, we only tested vestibular stimulation to enhance sleep, leaving it unclear if other sensory stimulation approaches (i.e., acoustic stimulation) would be similarly effective. For chronic experiments, sleep was quantified using a motion detection algorithm that defined sleep as any immobile period exceeding 40 s. This approach may have misclassified rare periods of quiet wakefulness lasting over 40 s as sleep.

Finally, we did not investigate the specific—and likely multiple—mechanisms through which sleep enhancement influences AD pathology. Further research is essential to elucidate the various pathways by which improved sleep may impact AD progression.

## Resource availability

### Lead contact

Requests for further information and resources should be directed to and will be fulfilled by the lead contact, Michele Bellesi (michele.bellesi@unicam.it).

### Materials availability

This study did not generate new unique reagents.

### Data and code availability

• Data: All data reported in this paper will be shared by the lead contact upon request.
• Code: This paper does not report original code, but the analysis scripts are available (https://github.com/BSRLab).
• Additional information: Any additional information required to reanalyze the data reported in this paper is available from the lead contact upon request.

## Acknowledgments

We thank Amina Aboufares El Aloui for initial technical support. This work was supported by Alzheimer Research UK (ARUK PPG2020A-023 to MB), 10.13039/100010269Wellcome Trust (215267/Z/19/Z to MB), 10.13039/100006781Giovanni Armenise-Harvard Foundation (CDA for L.d.V.), the 10.13039/501100000780European Union – Next generation EU, Ministry of University and Research-Promotion and Development Fund- Ministerial Decree N737/2021 -MYSLEEP (L.d.V.).

## Author contributions

Conceptualization: M.B. and L.d.V.; investigation: L.Z., L.S., N.A.N., R.S., E.F., L.d.V., and M.B.; writing—original draft: L.Z., L.S., L.d.V., and M.B.; writing—review and editing: all authors.; project supervision and funding: M.B. and L.d.V.

## Declaration of interests

The authors declare no competing interests.

## Declaration of generative AI and AI-assisted technologies in the writing process

The authors utilized ChatGPT 4.0 during the revision of this work to assist with grammar and spelling corrections. Following its use, the authors thoroughly reviewed and edited the content as necessary and accepted full responsibility for the final published version of the article.

## STAR★Methods

### Key resources table

**Table:** 

REAGENT or RESOURCE	SOURCE	IDENTIFIER
**Antibodies**		

Anti-Beta-Amyloid 1-42 Antibody	Millipore	Cat# AB5078P; RRID: AB_91677
Anti-tau antibody	Sigma-Aldrich	Cat# T9450; RRID: AB_477595
Anti-pTau (AT8) antibody	Thermo Fisher Scientific	Cat# MN1020; RRID: AB_223647
Anti-pTau-s404	Abcam	Cat# ab196364
Anti-Rabbit HRP conjugated	Millipore	Cat# 12-348; RRID: AB_390191
Anti-Mouse HRP conjugated	Cell Signaling Technologies	Cat# 7076S; RRID: AB_330924

**Chemicals, peptides, and recombinant proteins**		

DL-Dithiothreitol (DTT)	Sigma-Aldrich	Cat# D9163
EGTA	Millipore	Cat# 324626
HEPES	Sigma-Aldrich	Cat# S-H0887
EDTA	Santa-Cruz	Cat# SC203932
PMSF	Sigma-Aldrich	Cat# 93482
Microcystin-LR	Sigma-Aldrich	Cat# 33893
EDTA-free Protease Inhibitor Cocktail	Roche	Cat# 04693159001
Sodium Dodecyl Sulfate (SDS) 10%	Bio-Rad	Cat# 1610416
Mini-PROTEAN TGX Gels	Bio-Rad	Cat# 4561095
10x Tris/Glycine/SDS Buffer	Bio-Rad	Cat# 1610772
10x Tris/Glycine Buffer	Bio-Rad	Cat# 1610771
Methanol	Carlo Erba	Cat# 414815
Tween 20	ITW Reagents	Cat# A4974
Nitrocellulose membrane	Bio-Rad	Cat# 1620215
Ponceau S stain	G-Biosciences	Cat# 279P-A
Methoxy-XO4	Biozol	Cat# HB5252

**Critical commercial assays**		

BCA Protein Assay Kit	Thermo Fisher	Cat# 23227
Chemiluminescence Kit	Amersham	Cat# PK-6100

**Experimental models: Organisms/strains**		

B6;129-Tg (APPSwe, tauP301L)1Lfa Psen1 tm1Mpm/Mmjax - 3xTg mice	Jackson Laboratories	RRID:MMRRC_034830-JAX

**Software and algorithms**		

Open Ephys	Open Ephys	open-ephys.org
MATLAB	R2022a	mathworks.com
ImageLab 5.0	Bio-Rad	bio-rad.com
Prism	GraphPad	graphpad.com
SleepSign-Sleep Stage Analysis	SleepSign	sleepsign.com

**Other**		

Orbital Shaker	IKA	HS260
Open Ephys acquisition board	Open Ephys	OEPS-9030

### Experimental model and subject details

#### Experimental animals

Five-week-old B6;129-Tg (APPSwe, tauP301L)1Lfa Psen1 tm1Mpm /Mmjax (3xTg, weight 25–35 g) male mice were acquired from the Jackson Laboratory (Bar Harbor, ME USA 04609). All mice were housed in groups of four in environmentally controlled cages and subsequently placed in sleep recording chambers. Mice were under 12 h dark/light cycle with lights on at 8:00 P.M., at the temperature of 24 ± 1°C, with food and water available *ad libitum* and replaced daily at 8:00 A.M. All procedures were in accordance with the guidelines laid down by the European Communities Council Directives (2010/63/EU) for the care and use of laboratory animals under an approved protocol (609/2023-PR) by Veterinary Health Dept. of the Italian Ministry of Health. All appropriate measures were taken to minimize pain and discomfort in experimental animals.

### Method details

#### Experimental design

Male mice were divided into two weight-balanced groups, the sleep enhancement (SE) and normal sleep (S) group. SE was achieved by placing the animals, housed in a open top plexiglass cage, on a reciprocating linear-motion shaker (HS 260 Control, IKA, Switzerland). This shaker generated motion along a single axis in the horizontal plane with a fixed maximum displacement of ±20 mm at 1.0Hz periodicity. It is important to note that 1Hz frequency corresponds to the harmonic motion of the shaker itself and not the encoded vestibular stimulus, which may occur at twice the shaker’s rocking rate.^27^ In previous work, this procedure has been shown to increase the time spent in NREM sleep at the expenses of wake time.^27^ Mice in the S group were positioned within the identical sleep apparatus used for the SE mice. However, they remained undisturbed with the rocking system turned off. Rocking started following a 10-day acclimation period, with the final three days serving as the baseline. In a first set of experiments (Exp 1, n=16, 8 [SE] + 8 [S]) aimed at determining the optimal duration of the chronic stimulation, SE mice were rocked during the light period for 12 hours for 20 consecutive days. In the second experiment (Exp 2, n=24, 12 [SE] + 12 [S]), we opted for a different paradigm of stimulation by introducing a block design (one week of stimulation always during the light period only followed by one week of no stimulation for nine cycles – 18 weeks overall, starting at P60). In the third experiment (Exp 3, n=12, 6 [SE] + 6 [S]), mice were subjected to the same block design as in Exp 2, but the duration of the experiment was limited to the first two cycles. Throughout the whole Exp 2, mice were behaviorally evaluated biweekly using hindlimb clasping test and ledge walking test.

#### Polysomnographic recordings

A subset of mice (n=6) was implanted with electrodes for electroencephalographic recordings and was used to test the efficacy of the rocking procedure in this mouse model. Briefly, mice were anesthetized with isoflurane (2.5% for induction and 1.5% for maintenance) and implanted with frontal and parietal electrodes to monitor brain activity. An additional electrode was placed above the cerebellum and used as reference. To measure muscle activity, a pair of silver wires were placed into the muscles on both sides of the back of the neck. Implanted mice were kept individually in transparent Plexiglas cages throughout the experiment. After recovery from the surgery, recordings started at the beginning of the light phase. Data acquisition was carried out continuously with an Open Ephys system across the 24h per 2 days. The signal was filtered at 0.1-40 Hz for EEG and 10-50 Hz for EMG. The paradigm of acquisition consisted in 1 day of baseline and one day of sleep enhancement via rocking at 1Hz during the light phase. EEG scoring was performed offline by visual inspection of 4s epochs using Sleep Sign software. Behavioral state and power analysis were analyzed using custom-made MATLAB scripts. Slow wave activity (SWA) was computed by averaging the EEG NREM sleep power spectrum between 0.5 and 4Hz. Cumulative SWA (slow wave energy, [SWE]) was calculated by cumulative summing NREM sleep SWA over 12h of the light period. Sleep episodes were defined as periods of NREM sleep longer than 8 seconds. Short periods of wake shorter than 16 seconds in between NREM sleep episodes of at least 20 seconds were identified as brief arousals. Beta power was computed by averaging the EEG NREM sleep power spectrum between 15 and 30Hz.

#### Sleep quantification using motion detection

To avoid possible tissue damage and inflammation resulting from the implant of EEG electrodes, in chronic experiments sleeping and waking states were inferred by the analysis of motion activity obtained by continuous video monitoring with infrared cameras. Using this method, we defined sleep as periods of immobility lasting ≥ 40 seconds but could not differentiate between NREM and REM sleep. This approach has been previously validated with EEG-implanted animals, demonstrating a high concordance rate (93% on average across eight mice) between motion-based and EEG-based sleep/wake estimations.^52^^,^^53^ Similar validation has also been reported in other studies.^54^ In designing the shaking apparatus, we ensured that cage movement did not interfere with motion detection. The cameras were mounted on top of the cage, and they moved in sync with the cage during rocking, allowing the detection algorithm to accurately track mice movements without introducing artifacts from the cage relative motion within its environment.

To assess sleep fragmentation, we calculated a sleep fragmentation index based on motion activity, defined as the number of transitions from sleep to wakefulness per hour.

Both sleep amount and the sleep fragmentation index for each mouse were normalized to baseline data collected during the acclimation period. This normalization was essential to account for potential variability in sensitivity across different video cameras, enabling pooled analysis of the data. Normalization also improved the possibility to detect small effect sizes while minimizing the number of mice required for the study.

#### Ledge walking and hindlimb clasping test

The ledge walking was used to test motor coordination, which is usually impaired in mice models of neurodegenerative disorders. The test was performed by placing a mouse on the cage’s ledge. Mice typically walk along the ledge and attempt to descend back into the cage. We assigned a test score (0 to 3) based on walking posture. Normal (score 0); slight imbalance while walking (score 1); ineffectively use of hind legs or landing on the head (score 2); falling off or extreme reluctance to move (score 3).

Hindlimb clasping is a simple test that assesses hindlimb position relative to the body and is used to evaluate disease progression in mouse models of neurodegenerative diseases.^44^^,^^55^ The mice were lifted by grasping the tail near its base and observed for 10 seconds. A score (0 to 3) was given as it follows: hindlimb consistently splayed outward (score 0), one hindlimb retracted >5 s of the time (score 1), both hindlimbs retracted >5 s (score 2), both hindlimbs fully retracted and touch the abdomen >5 s (score 3). Each of these tests was conducted three times, and the average score was calculated for each time point.

#### Novel object recognition

After the 18 weeks of sleep manipulation, mice were tested with a novel-object recognition (NOR) test. NOR was performed in an open field with a camera mounted above the open field recorded the movements of the mouse throughout the trial. The NOR test had three phases: the habituation phase, where mice freely investigate the box for 5 minutes; the training phase, involving a 10-minute exposure to a box with two identical objects; and the testing phase, occurring after a 3-hour intertrial pause, where mice explore a box containing one novel and one known object for 10 minutes. The box was cleaned using 75% ethanol between each trial. The time spent exploring each object a discrimination score was calculated with the following formula: discrimination score = (time exploring the novel object − time exploring the familiar object)/total time exploring both objects.

#### Tissue collection

The next day following the NOR test or the end of sleep manipulation (Exp 3), mice intended for molecular work (n=22, 12 [S] + 12 [SE]) were sacrificed between 9:00 and 11:00 A.M. to maintain the tissue collection within the same 2-h time of day window for all experimental groups. Mice were briefly anesthetized with isoflurane (1–1.5% volume) and sacrificed by cervical dislocation. Brains were extracted, quickly dissected to separate the cortex, were immediately frozen in dry ice, and were used for molecular evaluations. Mice intended for methoxy-X04 staining (n=8, 4 [S] + 4 [SE]) were perfused intracardially with a solution containing phosphate buffer and paraformaldehyde 4%. Brains were post-fixed for 24 hours in the same fixative and then sliced in 50 μm coronal sections using a vibratome. Of note, mice used for methoxy-X04 staining were not video monitored and their motion activity was not recorded.

#### Western blotting

Through a glass/glass tissue homogenizer, mouse cortex tissue samples were homogenized in freezing homogenization buffer containing 10 mM HEPES, 1.0 mM EDTA, 2.0 mM EGTA, 0.5 mM DTT, 0.1 mM PMSF, Protease Inhibitor Cocktail, 100 nM microcystin. Next, 500 μL of each sample was boiled in 50 μL of Sodium Dodecyl Sulfate (SDS) at 90/95°C for 8 min and kept at – 80°C. The protein concentration was evaluated with bicinchoninic acid assay. One sample was discarded for technical problems during the preparation.

Equal amounts of protein from each animal (15μg for beta-amyloid, 5μg for total tau, and 14 μg for p-tau) were loaded onto the same gels with sample loading order randomized. Homogenate samples of each S and SE mice were resolved by Tris-HCl gel electrophoresis in 1X Tris/Glycine/SDS running buffer. Then, the proteins were transferred onto 0.45 μm pore size nitrocellulose membranes in 1X Tris/Glycine/Methanol transfer buffer. After transfer, membranes were stained with Ponceau S, acquired for total protein quantification at the Chemidoc, and then washed 3 times with phosphate buffer solution (PBS) + 0.1% Tween-20. The membranes were immunoblotted as follows. First, they were blocked in 5% non-fat dry milk in PBS + 0.1% Tween-20 with gentle shaking for 1h at room temperature; then, they were incubated overnight at 4°C with one of the following primary antibodies: anti-beta-amyloid 1-42 antibody (beta-amyloid, 1:1000), anti-tau antibody, mouse monoclonal (TAU, 1:2000) or anti-pTau antibody (AT8 [1:1000] or pTau-s404 [1:1000]) diluted in PBS + 0.1% Tween-20 with 5% bovine serum albumin. Next, membranes were rinsed 3 times in PBS + 0.1% Tween-20 and then incubated with one of the following secondary antibodies: goat anti-rabbit IgG (1:10000 for anti-beta amyloid 1-42 and 1:2000 for pTau-s404) or Anti-mouse IgG (1:5000), HRP-linked antibody diluted in PBS + 0.1% Tween-20 with 3% non-fat dry milk for 90 min at room temperature with mild oscillation. Membranes were washed 3 times in PBS + 0.1% Tween-20 and, in the end, with ddH2O, incubated with enhanced ECL Chemiluminescence Reagent, and the bands were revealed by exposition to Molecular Imager ChemiDoc XRS+ (Bio-Rad). The optical density of the bands was quantified by the Image Lab 5.0 software (Bio-Rad). Since housekeeping proteins (e.g., β-actin and β-tubulin) can be affected by sleep and wake, they were not used as internal standard. Instead, optical density values were normalized to total protein loading obtained by the ponceau S staining ^56^ (normalized experimental signal = observed experimental signal/lane normalization factor; the lane normalization factor = observed signal of total protein for each lane/by the highest observed signal of total protein on the blot).

#### Methoxy-X04 staining

Sections from both experimental conditions were washed with phosphate buffer and then exposed to Methoxy-X04 (1μg/ml) for 30 minutes to highlight beta-amyloid plaques. After staining sections were washed again, mounted on microscopy slides, and imaged using a confocal microscopy. Multiple random fields of cerebral cortex and hippocampus were analyzed for both groups of mice.

### Quantification and statistical analysis

Statistical analysis was performed using GraphPad Prism software (La Jolla, CA, USA) and MATLAB (The MathWorks Inc., Natick, MA, USA). Parametric statistics were used, alpha was set to 0.05 and appropriately corrected for multiple comparisons when required. A summary of all the statistical comparisons and results is reported in Table S1.
