# Supplementary material for: Rocking during sleep reduces motor deficits and beta-amyloid levels in an Alzheimer’s mouse model
Source: iScience. 2025 Feb 15;28(3):112036. doi: 10.1016/j.isci.2025.112036 (PMC11925102; doi:10.1016/j.isci.2025.112036)
Supplement: Document S1. Figure S1 and Table S1 [file mmc1.pdf]

iScience, Volume *28*

Supplemental information

Rocking during sleep reduces motor deficits and beta-amyloid levels in an Alzheimer's mouse model

Luyan Zhang, Letizia Santoni, Nam Anh Ngo, Reyila Simayi, Eleonora Ficiará, Luisa de Vivo, and Michele Bellesi

SUPPLEMENTARY MATERIAL

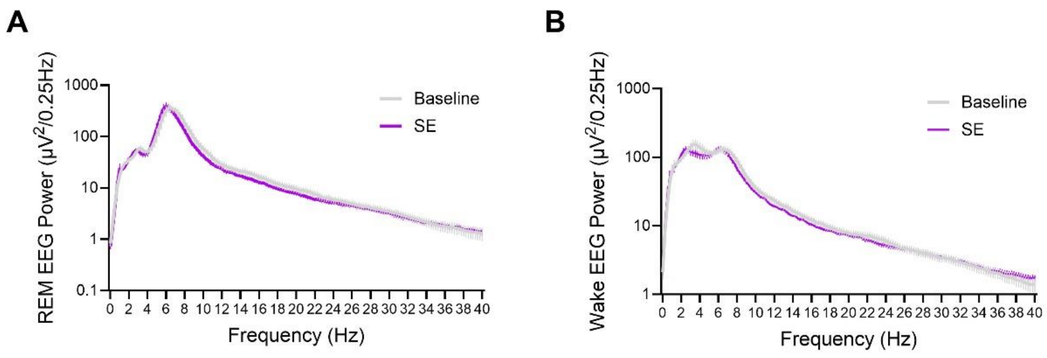

Figure S1. Effects of rocking on REM and Waking power spectra. A-B. Absolute REM sleep (**A**) and waking (**B**) power spectrum of BSL and SE (n=6, values are mean ± sem).

Table S1. Summary of statistical results

	Parameter	Results	Statistical Test
**Figure 1**			
Sleep architecture 24h	NREM, REM, W time	No effect	2-way ANOVA F (1, 15) = 2.516e-014 P>0.9999
Sleep architecture light period	NREM, REM, W time	↑ NREM sleep	2-way ANOVA Vigilant state x condition F (2, 15) = 59.23 P<0.0001.<br>Šídák's multiple comparisons test<br>W: P < 0.0001<br>NREM: P < 0.0001<br>REM: P = 0.99
Sleep architecture dark period	NREM, REM, W time	No effect	2-way ANOVA: Vigilant state x condition F (2, 15) = 0.2641 P = 0.7714<br>Šídák's multiple comparisons test<br>W: P = 0.92<br>NREM: P = 0.98<br>REM: P = 0.99
Distribution of NREM episode duration	Number of NREM episodes	↑ NREM sleep episode (0-40 s)	2-way RM ANOVA condition F (1, 80) = 8.781 P = 0.0040<br>Šídák's multiple comparisons test<br>0-20 s: P = 0.0597<br>20-40 s: P < 0.0001
NREM time course	NREM sleep amount	↑ NREM sleep episode (0-2 h)	2-way RM ANOVA condition F (1, 60) = 10.18 P = 0.0023<br>Šídák's multiple comparisons test<br>0-2: P < 0.0001<br>2-4: P = 0.0789
**Figure 2**			
NREM SWA time course (24h)	NREM SWA amount	No effect	2-way RM ANOVA condition F (1, 60) = 1.474e-013 P>0.9999
NREM SWA time course (12h light period)	NREM SWA amount	No effect	2-way RM ANOVA condition F (1, 30) = 2.751 P = 0.1076
NREM SWE	SWE	↑ in SE	Paired t-test: P = 0.0107
NREM Absolute spectrum	Power	No effect	2-way RM ANOVA condition F (1, 10) = 1.000 P = 0.3409<br>Frequency x condition F (160, 1600) = 2.16 P < 0.0001

NREM Absolute spectrum (0.5-4 Hz)	Power	↓ in SE	2-way RM ANOVA NREM: frequency x condition F (14, 140) = 4.691 P < 0.0001<br>Šídák's multiple comparisons test:<br>[bin 3-3.25 Hz]: P = 0.0285<br>[bin 3.25-3.5 Hz]: P = 0.026
NREM Absolute spectrum (0.5-4 Hz) – dark period	Power	No effect	2-way RM ANOVA NREM: frequency x condition F (4, 140) = 0.34, P = 0.99
Brief arousals	Density n./h	No effect	Paired t-test: P = 0.53
Beta power	Mean power	No effect	Paired t-test: P = 0.19
**Figure 3**			
Sleep amount light period	Sleep amount	↑ in SE	2-way ANOVA sleep amount: condition F (1, 280) = 17.66 P < 0.0001<br>Šídák's multiple comparisons test:<br>Day1: P = 0.0006<br>Day2: P = 0.04
Sleep amount dark period	Sleep amount	No effect	2-way ANOVA sleep amount: condition F (1, 280) = 2.547 P = 0.1116
**Figure 4**			
Sleep amount light period through days	Sleep amount	↑ in SE	1-way RM ANOVA F (2.214, 15.50) = 2.078 P = 0.1556<br>Uncorrected Fisher's LSD<br>BSL vs Day1: P = 0.0069<br>BSL vs Day2: P = 0.019
Sleep amount dark period through days	Sleep amount	No effect	1-way RM ANOVA F (1.989, 13.92) = 0.95 P = 0.4096
Sleep amount light period through cycles	Sleep amount	↑ in SE	1-way RM ANOVA F (1.732, 10.39) = 7.995 P = 0.0094<br>Dunnett's multiple comparisons test<br>BSL vs Cycle1: P = 0.037<br>BSL vs Cycle2: P = 0.0057
**Figure 5**			
Sleep fragmentation index through days	Sleep fragmentation index	↓ in SE	1-way RM ANOVA F (2.873, 20.11) = 3.282 P = 0.0436<br>Uncorrected Fisher's LSD<br>BSL vs Day1: P = 0.0117
Sleep fragmentation index through cycles	Sleep fragmentation index	↓ in SE	1-way RM ANOVA F (3.024, 18.15) = 5.155 P = 0.0093<br>Dunnett's multiple comparisons test<br>BSL vs Cycle1: P = 0.0137
**Figure 6**			
Hindlimb clasping test	Score	No effect	2-way ANOVA F (1, 210) = 0.2307 P = 0.63

Ledge walking test	Score	↓ in SE	2-way ANOVA $F (1, 210) = 9.527$ $P = 0.0023$
	Averaged score (1 → 9)	↓ in SE	Unpaired t-test: $P = 0.004$
NOR	Discrimination Index	No effect	Unpaired t-test: $P = 0.54$
**Figure 7**			
beta-amyloid 1-42 (cortex)	Expression intensity	↓ in SE	Unpaired t-test: $P = 0.005$
Total Tau (cortex)	Expression intensity	No effect	Unpaired t-test: $P = 0.25$
AT8 (cortex)	Expression intensity	No effect	Unpaired t-test: $P = 0.26$
p-Tau (s404) (cortex)	Expression intensity	No effect	Unpaired t-test: $P = 0.58$
**Figure 8**			
beta-amyloid 1-42 (hippocampus)	Expression intensity	↓ in SE	Unpaired t-test: $P = 0.014$
Total Tau (hippocampus)	Expression intensity	No effect	Unpaired t-test: $P = 0.55$
AT8 (hippocampus)	Expression intensity	No effect	Unpaired t-test: $P = 0.34$
p-Tau (s404) (hippocampus)	Expression intensity	No effect	Unpaired t-test: $P = 0.42$
**Figure 9**			
Sleep amount through days	Sleep amount	↑ in SE	2-way ANOVA $F (1, 63) = 22.06$ $P < 0.0001$<br>Šídák's multiple comparisons test<br>BSL vs Day1: $P = 0.0002$<br>BSL vs Day2: $P = 0.009$
Sleep amount through cycles	Sleep amount	No effect	Two-way ANOVA $F (1, 8) = 0.01656$ $P = 0.9008$
beta-amyloid 1-42 (cortex)	Expression intensity	↓ in SE	Unpaired t-test: $P = 0.019$
Total Tau (cortex)	Expression intensity	No effect	Unpaired t-test: $P = 0.77$
Supplementary material			
**Supplementary figure 1**			
REM Absolute spectrum	Power	No effect	2-way ANOVA REM: Frequency x condition $F (160, 1600) = 0.74$, $P = 0.99$
Wake Absolute spectrum	Power	No effect	2-way ANOVA Wake: Frequency x condition $F (160, 1600) = 0.82$, $P = 0.94$
